# *RSC Advances* Outstanding Student Paper Awards 2024

**DOI:** 10.1039/d5ra90115d

**Published:** 2025-11-05

**Authors:** Laura Fisher

## Abstract

We are delighted to announce the winners of the *RSC Advances* Outstanding Student Paper Awards 2024. These awards recognise outstanding work published in the journal, in which a substantial component of the research was conducted by a student.
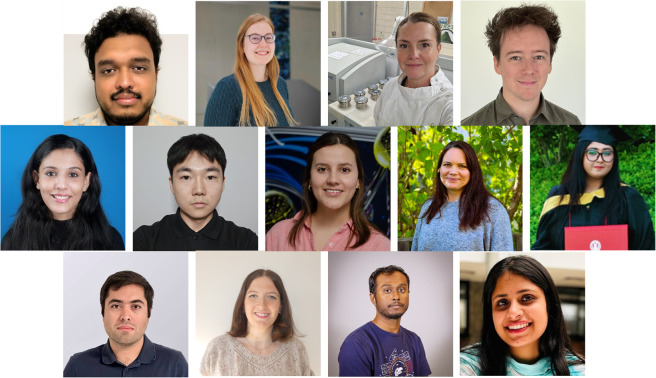

We are delighted to announce the winners for the *RSC Advances* Outstanding Student Paper Awards 2024. *RSC Advances* presents an annual award series to recognise the hard work of students within the chemistry community. All research articles published in *RSC Advances* in 2024 were considered. In order to be eligible for this award, the first author or co-first author must have been a student at the time of carrying out the research. From the support of corresponding authors, we received over 860 nominations highlighting the incredible talent and potential of emerging researchers in the field of chemistry. These awards continue to celebrate the exceptional work across a range of research fields and countries, reflecting the talent, diversity, and scientific curiosity that define the next generation of chemists.

The nominations were shortlisted based on several criteria, and the winning papers were then selected by our Editorial Board and Associate Editors (Table 1).

**Table 1:** Winners of the *RSC Advances* Outstanding Student Paper Awards 2024

*Analytical Chemistry*	Zhaokang Zhang, Fuzhou University, China
*Biological and Medicinal Chemistry*	Morgane Baudoin, Université Grenoble Alpes, France
*Catalysis*	Anjana Rajeev, National Institute of Technology Calicut, India
*Computational & Theoretical Chemistry*	Reza Ghanavati & Alma C. Escobosa, New Mexico State University, USA
*Energy Chemistry*	A M Mahmudul Hasan, University of Dhaka, Bangladesh
*Environmental Chemistry*	Melanie Maddin, Trinity College Dublin, Ireland
*Inorganic Chemistry*	T. Harri Jones, University of New Brunswick, Canada
*Materials Chemistry*	Vaishali Rathi, University of Petroleum and Energy Studies UPES, India
*Nanoscience*	Christina Wenck, Fraunhofer Institute for Microengineering and Microsystems IMM, Germany
*Organic Chemistry*	Mintu Munda, Indian Institute of Science Education and Research Bhopal, India
*Physical Chemistry*	Maria Bånkestad, Uppsala University, Sweden
*Food Chemistry*	Nafisa Sadaf, University of Arkansas, USA

Below, we highlight the winner of each subject category, and the research paper that won them the award. Please join us in congratulating all our winners for their exceptional achievement. We look forward to witnessing their continued growth and impact as they embark on a promising career in the field of chemistry.

## Analytical Chemistry

**Zhaokang Zhang, Fuzhou University, China** 
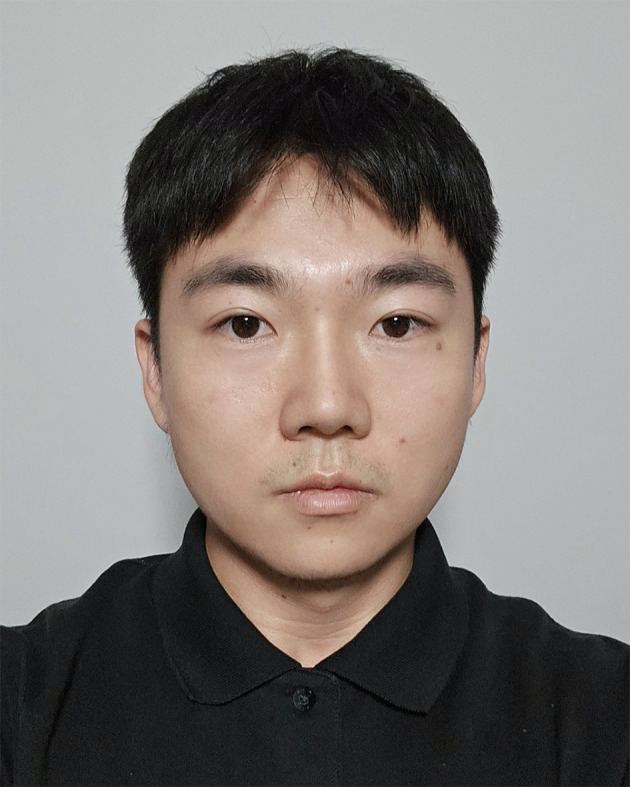


Zhaokang is recognised for his outstanding contribution to the research presented in:

*Portable glucose sensing analysis based on laser-induced graphene composite electrode* (https://doi.org/10.1039/D3RA06947H)

Zhaokang Zhang has a Master’s degree in materials and chemical engineering from Fuzhou University under the supervision of Professor Li Yanxia. His research focuses on enzyme electrochemical sensors, where he specializes in enhancing micro-sensing electrode performance through advanced materials engineering while capitalizing on enzymatic bioelectrocatalysis to develop next-generation flexible wearable biosensors.

Prior to his graduate studies, he earned a Bachelor’s degree in materials science from Hefei University of Technology in 2021. Building upon his expertise in polymer synthesis and nanomaterial fabrication, he is presently engaged in cutting-edge research dedicated to optimizing high-performance polyolefin production systems through catalytic process innovation and process intensification strategies.

## Biological and Medicinal Chemistry

**Morgane Baudoin, Université Grenoble Alpes, France** 
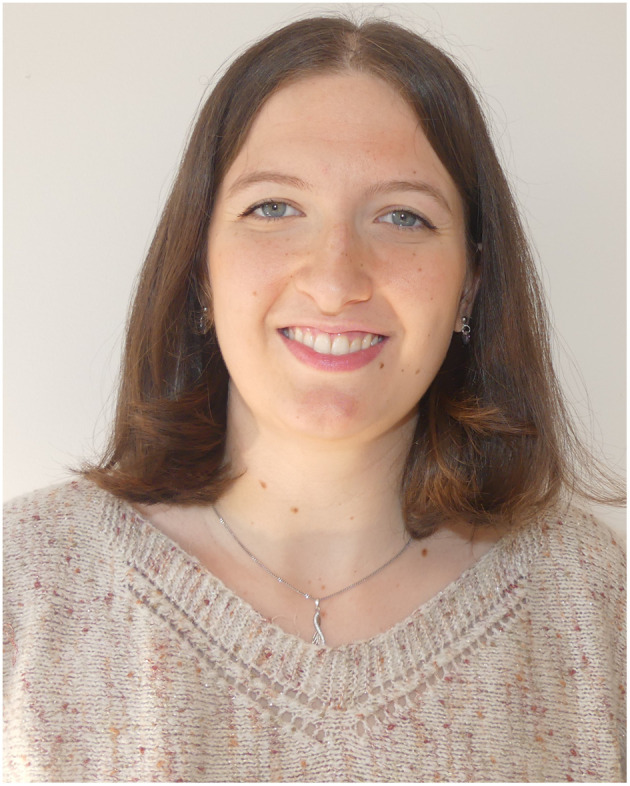


Morgane is recognised for her outstanding contribution to the research presented in:

*To click or not to click for short pulse-labeling of the bacterial cell wall* (https://doi.org/10.1039/D4RA04945D)

Morgane Baudoin obtained an engineering degree in chemistry in 2017 from the Graduate School of Chemical, Materials and Industrial Engineering (ENSIACET) in Toulouse, France. After six months of experience as an intern at the pharmaceutical company Servier, where she worked on the synthesis of polymer–drug conjugates (PDC), she pursued a PhD in chemical biology at the Department of Molecular Pharmacochemistry (DPM) in Grenoble under the supervision of Dr Yung-Sing Wong and graduated in 2023. Through the design and synthesis of novel organic compounds, she explored the metabolic incorporation of teichoic acid and peptidoglycan precursors in the cell wall of *Streptococcus pneumoniae* for labelling and therapeutic purposes. This work led to the publication of 4 research papers in peer-reviewed international journals and several oral communications at national and international conferences. She is currently working as a postdoctoral researcher at Uppsala University in Sweden with Dr Luke Odell.

## Catalysis

**Anjana Rajeev, National Institute of Technology Calicut, India** 
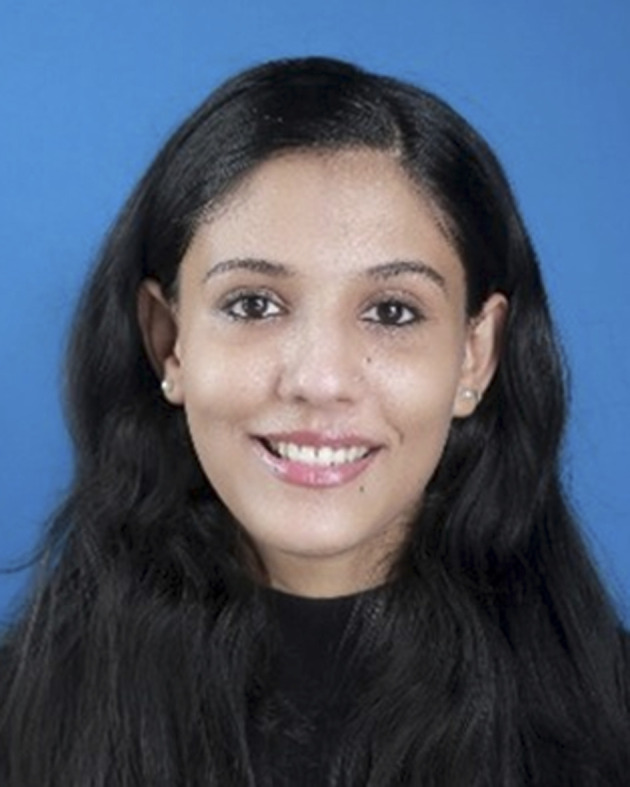


Anjana is recognised for her outstanding contribution to the research presented in:

*Selective synthesis of cyclic alcohols from cycloalkanes using nickel(* *ii* *) complexes of tetradentate amidate ligands* (https://doi.org/10.1039/D4RA05222F)

Anjana Rajeev K obtained her Master’s degree in applied chemistry from the University of Calicut. She then pursued her PhD at the National Institute of Technology Calicut, India, under the supervision of Dr Muniyandi Sankaralingam, and was awarded her degree in April 2025. Her doctoral research focused on the synthesis and characterization of diverse molecular nickel(ii) complexes and their catalytic applications in the oxidation of unactivated C–H bonds in substrates such as cycloalkanes and benzene derivatives. Her broader research interests include the generation, trapping, and reactivity studies of first-row transition metal–oxygen species.

## Computational & Theoretical Chemistry

**Reza Ghanavati & Alma C. Escobosa, New Mexico State University, USA** 
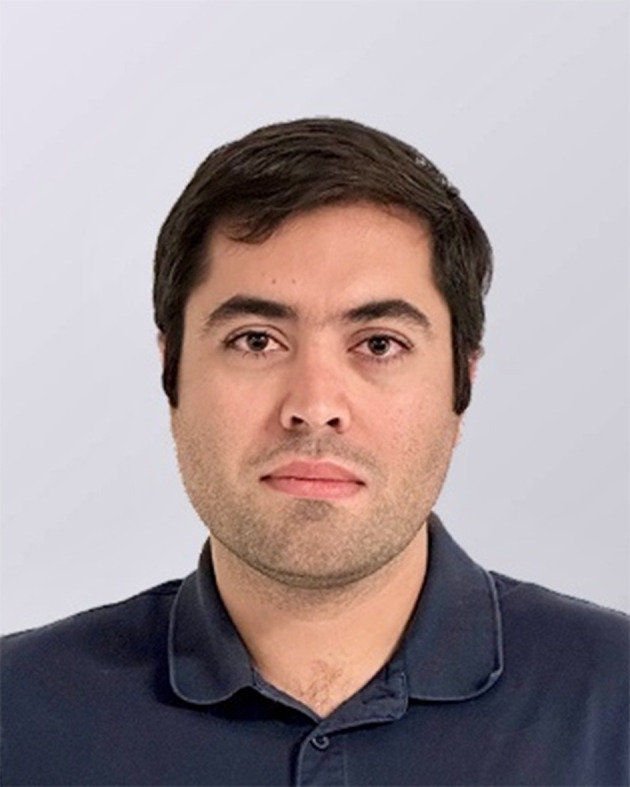
 
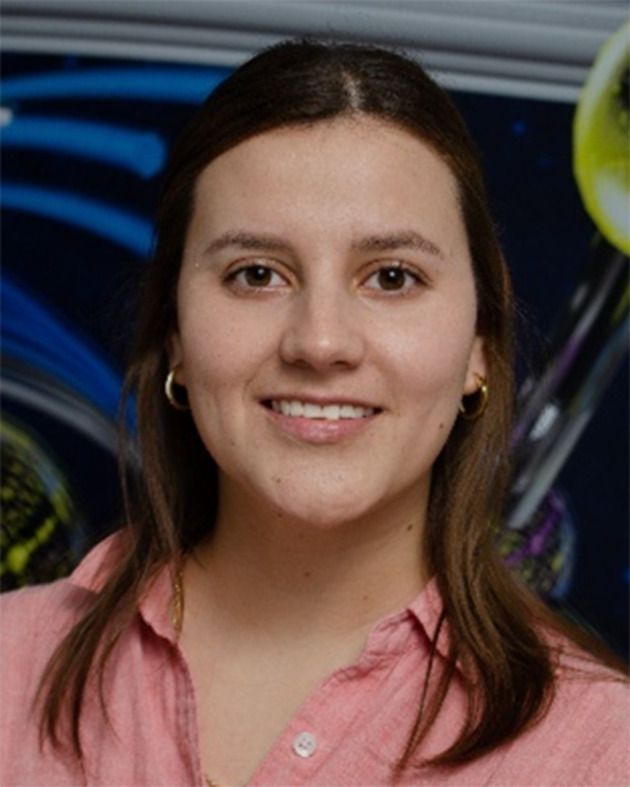


Reza & Alma are recognised for their outstanding contribution to the research presented in:

*An automated protocol to construct flexibility parameters for classical forcefields: applications to metal–organic frameworks* (https://doi.org/10.1039/D4RA01859A)

Reza Ghanavati is a fifth-year PhD student in chemical and materials engineering at New Mexico State University, where he conducts research under the mentorship of Dr Thomas A. Manz. His doctoral work focuses on developing automated methodologies for polarizable, flexible forcefields to study gas separations in metal–organic frameworks (MOFs), targeting hydrogen purification in solar water-splitting applications. He earned his Bachelor’s degree in Chemical Engineering from Amirkabir University of Technology and a Master’s degree in chemical engineering from Sharif University of Technology, specializing in simulation and control. Beyond his academic research, Reza has applied machine-learning algorithms and developed interactive R Shiny applications to advance pharmacometrics modelling, enhancing predictions of pharmacokinetics and pharmacodynamics (PK/PD) parameters. He has also gained industry experience through internships at leading pharmaceutical companies, including GSK, Bristol Myers Squibb, and Johnson & Johnson.

Alma C. Escobosa earned her Bachelor’s degree in chemistry from the University of Texas at El Paso in 2018. She then continued to pursue her PhD in chemical engineering at New Mexico State University, completing it in 2024. Her doctoral research, conducted under the supervision of Dr Thomas Manz, focused on developing interatomic potentials (forcefields) for metal–organic frameworks (MOFs) to allow prediction of helium separation performance from natural gas. Her dissertation presents a roadmap for constructing, validating, and applying forcefields to use in atomistic simulations. Along the way, she was a recipient of the Frontera Computational Science Fellowship and the CONACYT fellowship, both of which she is grateful for.

## Energy Chemistry

**A M Mahmudul Hasan, University of Dhaka, Bangladesh** 
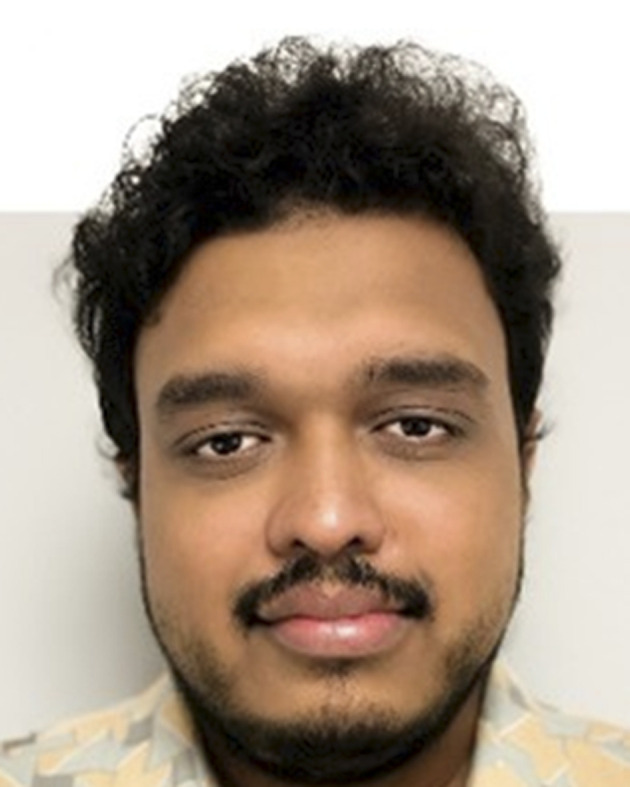


Mahmudul is recognised for his outstanding contribution to the research presented in:

*Synergism in carbon nanotubes and carbon-dots: counter electrode of a high-performance dye-sensitized solar cell* (https://doi.org/10.1039/D4RA00601A)

A M Mahmudul Hasan completed his BSc in chemistry (2021) and MSc in physical chemistry (2022) at the University of Dhaka, Bangladesh, where he conducted research in the Materials Chemistry Research Laboratory (MCRL) under the supervision of Prof. Md. Abu Bin Hasan Susan. Mahmudul’s research centres on the design and synthesis of π-conjugated, structurally ordered electronic materials with high electrical conductivity that are cost-effective and readily processable for next-generation energy technologies. Motivated by the urgent need for environmentally sustainable energy solutions, his Master’s research focused on developing composite carbon materials as counter electrodes to enhance the performance of dye-sensitized solar cells. This research led to fellowship awards from the Bangladesh National Institute of Science and Technology (NIST) and the Semiconductor Research Institute at the University of Dhaka. Mahmudul is currently pursuing a PhD in physical chemistry at the University of Florida, USA, where his work expands to the development of advanced organic polymer-based electronic materials. His doctoral research has been recognized with the Louise and V.T. Jackson Summer Fellowship and the Martin T. Vala Award for Research Excellence in Physical Chemistry. Mahmudul remains dedicated to advancing his research toward the development of novel, scalable, and economically viable materials that facilitate the transition to renewable energy technologies.

## Environmental Chemistry

**Melanie Maddin, Trinity College Dublin, Ireland** 
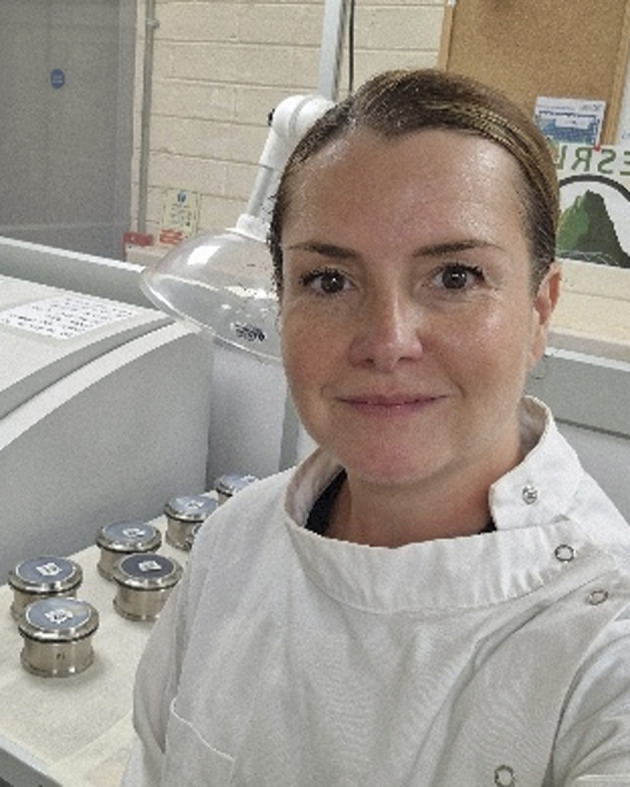


Melanie is recognised for her outstanding contribution to the research presented in:

*Transient crystallisation of rare earth carbonates during the hydrothermal oxidation of siderite* (https://doi.org/10.1039/D4RA05212A)

Melanie Maddin is a geochemist with a BSc in Earth science and a PhD in geochemistry from Trinity College Dublin. Her doctoral research explored how rare-earth elements are taken up and fractionated in carbonates, iron carbonates, and iron phosphates. She now works as a laboratory technician and research fellow in the Earth Surface Research Laboratory at Trinity College Dublin, where she supports and develops cutting-edge geochemical research. Her upcoming project will investigate carbon, nutrient, and trace-element dynamics in the coastal systems of North County Dublin, with the aim of advancing our understanding of how these environments respond to environmental change.

## Inorganic Chemistry

**T. Harri Jones, University of New Brunswick, Canada** 
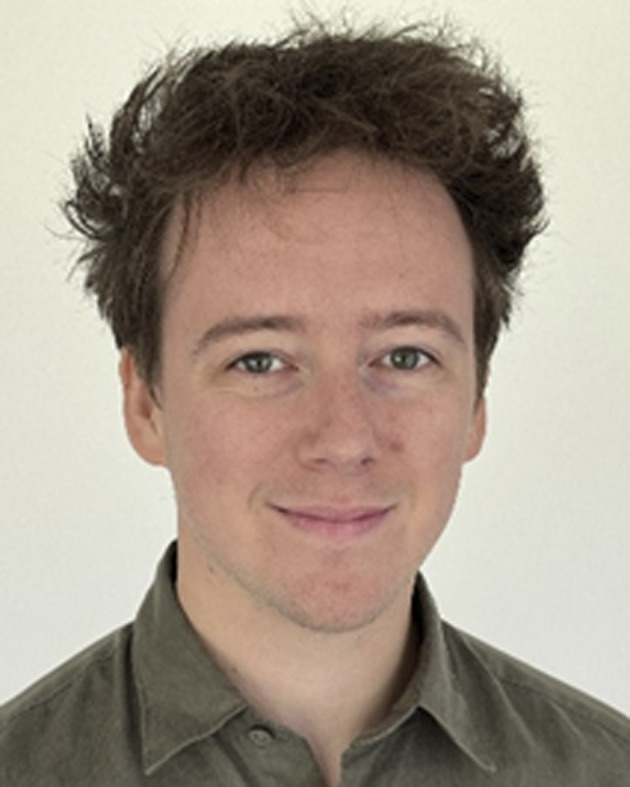


Harri is recognised for his outstanding contribution to the research presented in:

*Colour tuneability of heteroleptic iridium complexes through second-sphere coordination* (https://doi.org/10.1039/D4RA04535A)

Trystan Harri Jones obtained his MChem from the University of Edinburgh, Scotland in 2021. His Master’s thesis work was on the synthesis of amide-directed boron-doped polyaromatic hydrocarbons under the supervision of Prof. Michael Ingleson. He then went on to earn his PhD at the University of New Brunswick, Canada, in 2025 with the guidance of Prof. Barry Blight. His doctoral research centred on a large library of cyclometalated iridium complexes with tuneable optoelectronic properties that had potential applications from photocatalysts to emitters. The tuneable nature of these iridium complexes relied on complementary hydrogen-bonding arrays built into complexes which facilitate a change in emission in the presence of complementary H-bonding compounds.

During his PhD, Harri published multiple research papers in peer-reviewed journals, presented at several conferences (notably winning a poster prize at CANUK 2025), worked as a finalist judge for the Canada wide science fair, and delivered lectures in organic chemistry. Harri has accepted a post-doctoral research position with Prof. Catherine Aitchison at Linköping University, Sweden, in the laboratory of organic electronics.

## Materials Chemistry

**Vaishali Rathi, University of Petroleum and Energy Studies UPES, India** 
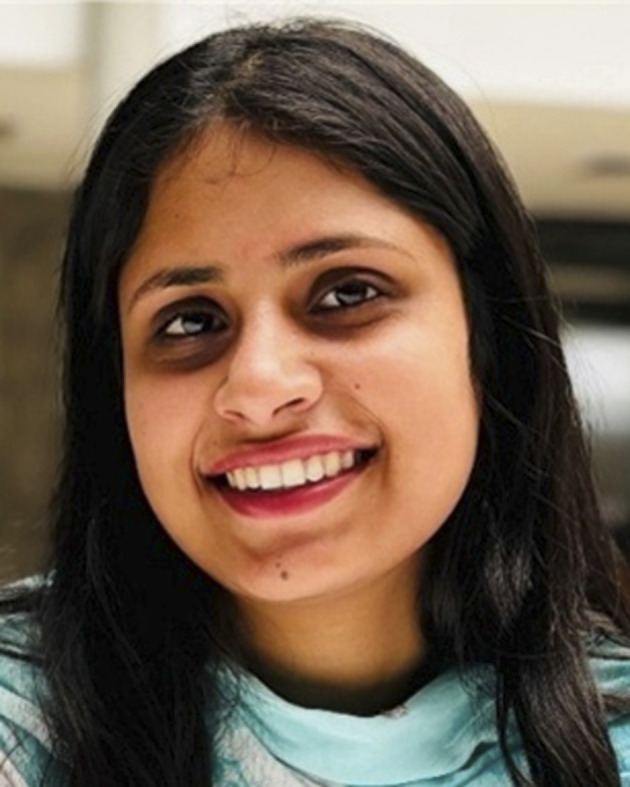


Vaishali is recognised for her outstanding contribution to the research presented in:

*Improved thermoelectric performance of PEDOT:PSS/Bi*_*2*_ *Te*_*3*_ */reduced graphene oxide ternary composite films for energy harvesting applications* (https://doi.org/10.1039/D4RA06184E)

Dr Vaishali Rathi is an INSPIRE Fellowship awardee with a strong academic and research background in materials science and nanotechnology. She completed her BSc (CBZ) from HNB Garhwal University, Srinagar, in 2015, followed by an MSc in organic chemistry from the same university in 2017, graduating with a Gold Medal for securing first rank.

She earned her PhD in materials science from the University of Petroleum and Energy Studies (UPES), Dehradun, in May 2025, under the supervision of Prof. Ashish Kumar and Prof. Ranjeet Kumar Brajpuriya. Her doctoral research focused on the design, synthesis, and multi-scale characterization of organic–inorganic hybrid composites for thermoelectric energy harvesting. She developed strategies to enhance the performance of graphene-based ternary composites with conducting polymers (PEDOT:PSS, PANI) and Bi_2_Te_3_, achieving improvements in electrical conductivity, Seebeck coefficient, and power factor.

Currently, Vaishali is a scientist at The Advance Carbon Company (TACC) Ltd., Madhya Pradesh, leading R&D on graphene materials for applications in concrete, textiles, and coatings. Outside research, she enjoys travelling and playing badminton.

## Nanoscience

**Christina Wenck, Fraunhofer Institute for Microengineering and Microsystems IMM, Germany** 
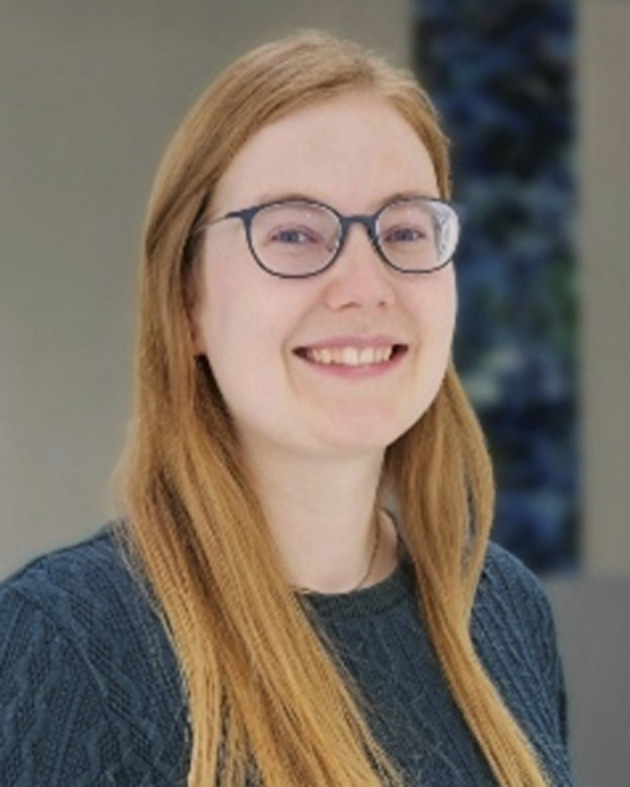


Christina is recognised for her outstanding contribution to the research presented in:

*Design and characterisation of casein coated and drug loaded magnetic nanoparticles for theranostic applications* (https://doi.org/10.1039/D4RA02626H)

Christina Wenck joined the Fraunhofer Institute for Microengineering and Microsystems (IMM) in Mainz, Germany, as a doctoral student in 2023, in the Nanomedicine group led by Dr Regina Bleul. Her research focuses on synthesizing magnetic nanoparticles using a continuous-flow micromixer setup, online analytics for real-time characterization, and developing magnetic nanoparticle systems for theranostic applications.

Christina holds Bachelor’s and Master’s degrees in nanotechnology from Leibniz University Hannover. After completing her Master’s degree in 2022, she worked in the Biophotonics group of Prof. Dr Heisterkamp at the Institute for Quantum Optics, Leibniz University Hannover. During this period, and while working on her Master’s thesis, her research focused on colorimetric detection of oral bacteria using functionalized gold nanoparticles as a plasmonic biosensor array, which was published in 2024 (https://doi.org/10.1039/D3NA00477E).

## Organic Chemistry

**Mintu Munda, Indian Institute of Science Education and Research Bhopal, India** 
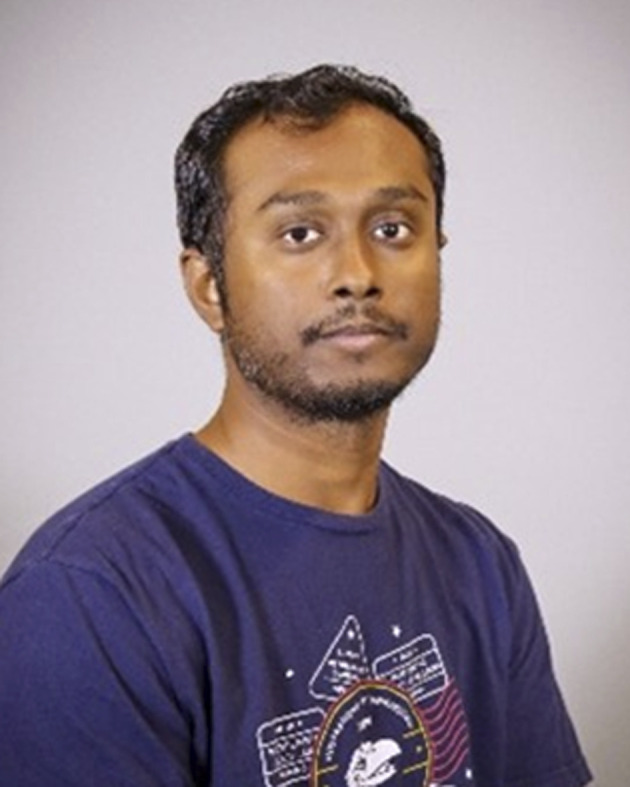


Mintu is recognised for his outstanding contribution to the research presented in:

*Total synthesis of naturally occurring abietane diterpenoids via a late-stage Fe(* *iii* *)-bTAML catalysed Csp* ^*3*^ *–H functionalization* (https://doi.org/10.1039/D4RA03791J)

Dr Mintu Munda, born in Kolkata, India, obtained his BSc (Hons.) in chemistry from Chakdaha College, affiliated to the University of Kalyani, in 2016. He then pursued an MSc in chemistry at the Indian Institute of Technology, Kharagpur. In 2018, Dr Munda joined the Indian Institute of Science Education and Research (IISER) Bhopal for his doctoral studies under the guidance of Prof. Alakesh Bisai, with Prof. Aasheesh Srivastava as co-supervisor. His PhD research focused on the total syntheses of indolosesquiterpene alkaloids, including xiamycins A and C–F, advancing synthetic strategies for biologically important natural products.

Following his PhD, Mintu joined the Department of Medicinal Chemistry at the University of Kansas, United States, in 2024 as a postdoctoral researcher in the group of Prof. Shyam Sathyamoorthi. His research focused on the development of catalytic asymmetric methods for the synthesis of β-amino acids and their application to biologically active frameworks.

In the same year, Mintu was awarded the prestigious Marie Skłodowska-Curie Postdoctoral Fellowship. He subsequently moved to the United Kingdom to join Queen Mary University of London (QMUL) as an MSCA postdoctoral researcher in the group of Dr Stellios Arseniyadis, where his current work explores biohybrid *N*-heterocyclic carbene (NHC) catalysis at the interface of synthetic methodology and chemical biology.

## Physical Chemistry

**Maria Bånkestad, Uppsala University, Sweden** 
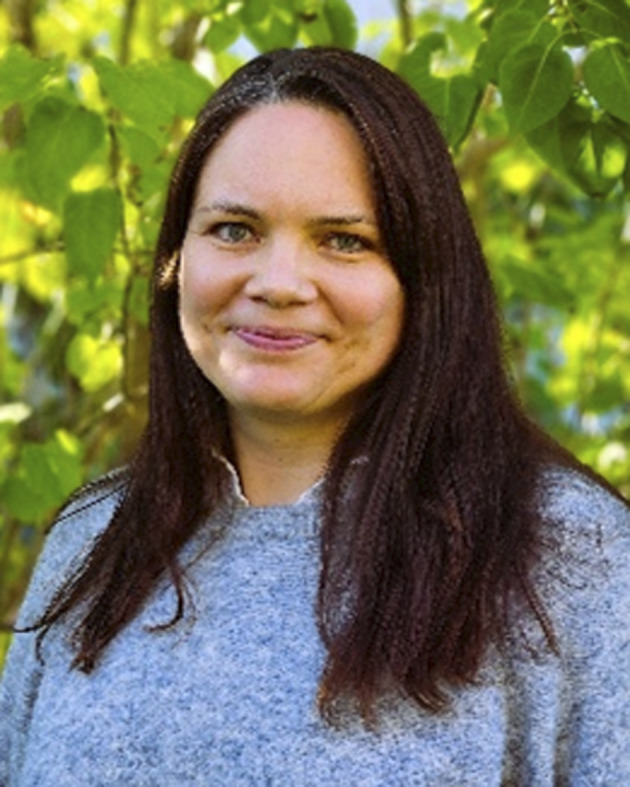


Maria is recognised for her outstanding contribution to the research presented in:

*Carbohydrate NMR chemical shift prediction by GeqShift employing E(3) equivariant graph neural networks* (https://doi.org/10.1039/D4RA03428G)

Maria Bånkestad defended her PhD in Machine Learning for Scientific Applications at Uppsala University in February 2025, with the dissertation *Structured models for scientific machine learning: From graphs to kernels*. Her doctoral work combined methods from graph neural networks and kernel approaches to tackle problems in the physical sciences, and her final paper was *Carbohydrate NMR chemical shift prediction by GeqShift employing E(3) equivariant graph neural networks*. Maria is currently a researcher at RISE Research Institutes of Sweden, where she applies machine learning to materials science and related fields.

## Food Chemistry

**Nafisa Sadaf, University of Arkansas, USA** 
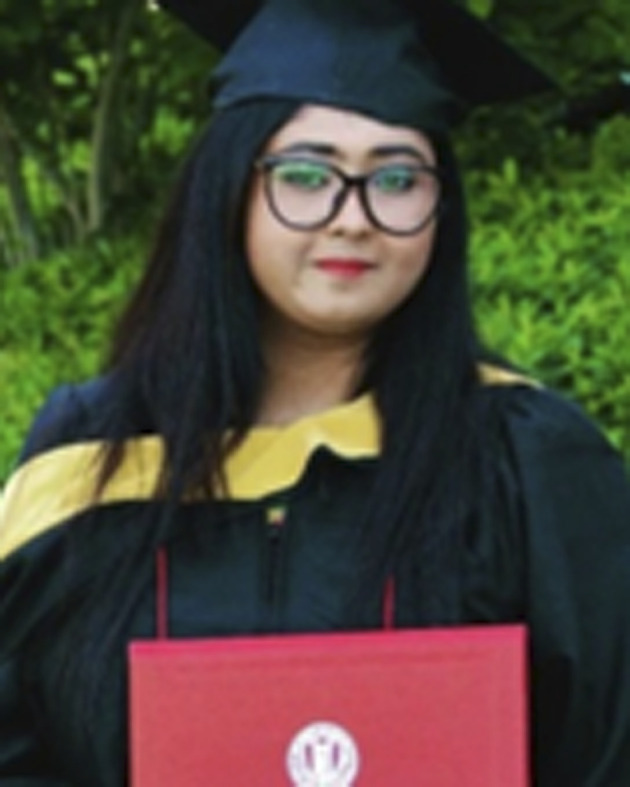


Nafisa is recognised for her outstanding contribution to the research presented in:

*Effect of a novel drying method based on supercritical carbon dioxide on the physicochemical properties of sorghum proteins* (https://doi.org/10.1039/D3RA07426A)

Nafisa Sadaf is a PhD student in food science at Florida State University under the supervision of Dr Leqi Cui. Her doctoral research focuses on enhancing the functionalities of plant proteins and evaluating their impact on the gut microbiome. She earned her MSc in food science at the University of Arkansas, working with Dr Ali Ubeyitogullari on improving sorghum protein properties using supercritical carbon dioxide (SC-CO_2_). Her thesis compared the physicochemical characteristics of SC-CO_2_-dried and freeze-dried sorghum proteins, providing novel insights into sustainable protein processing. Nafisa has published three peer-reviewed research papers and one conference proceeding. She was recognized as a finalist in the Protein and Co-Products Division Poster Competition at the AOCS Annual Meeting and Expo (2024). She completed her BSc in food engineering at Shahjalal University of Science and Technology, Bangladesh, where her undergraduate thesis explored edible coating development using ascorbic acid and aloe vera gel to extend fruit shelf life. Across her academic journey, Nafisa has consistently demonstrated a commitment to rigorous, application-oriented food science that advances sustainable protein systems and supports future innovations in nutrition and health.

Please join us in congratulating all our winners for their exceptional achievement. We extend our sincere gratitude to all the authors for their contributions, as well as to the editors and referees for their collaboration, which has resulted in this high-quality series.

We will continue to recognise outstanding student contributions and plan to give out these awards each year. If you published a research article in 2025, or go on to publish with the journal in the future, and would like to recognise a significant contribution made by a student, we invite them to join us in future editions of this series. Please email advances-rsc@rsc.org for more information.

Dr Laura Fisher, Executive Editor, *RSC Advances*

